# Denoising Diffusion Implicit Model Combined with TransNet for Rolling Bearing Fault Diagnosis Under Imbalanced Data

**DOI:** 10.3390/s24248009

**Published:** 2024-12-15

**Authors:** Chaobing Wang, Cong Huang, Long Zhang, Zhibin Xiang, Yiwen Xiao, Tongshuai Qian, Jiayang Liu

**Affiliations:** 1 State Key Laboratory of Performance Monitoring and Protecting of Rail Transit Infrastructure, East China Jiaotong University, Nanchang 330013, China; chaobing@163.com (C.W.); longzh@126.com (L.Z.); 2 School of Mechatronics and Vehicle Engineering, East China Jiaotong University, Nanchang 330013, China; conghuang@ecjtu.edu.cn (C.H.); xiangzb@ecjtu.edu.cn (Z.X.); evin@ecjtu.edu.cn (Y.X.); tongshuaiqian@ecjtu.edu.cn (T.Q.)

**Keywords:** rolling bearing, fault diagnosis, data imbalances, denoising diffusion implicit model, transformer

## Abstract

Data imbalances present a serious problem for intelligent fault diagnosis. They can lead to reduced diagnostic precision, which can jeopardize equipment reliability and safety. Based on that, this paper proposes a novel fault diagnosis method combining the denoising diffusion implicit model (DDIM) with a new convolutional neural network framework. First, the Gramian angular difference field (GADF) is used to generate 2D images, which are then augmented using DDIM. Next, by utilizing the weight-sharing properties of a convolutional neural network and the self-attention mechanism along with the global data processing capabilities of Transformers, a TransNet model is constructed. The augmented data are input into the model for training to establish a fault diagnosis framework. Finally, the method is validated and analyzed using the CWRU bearing dataset and the Nanchang Railway Bureau dataset. The results show that the proposed method achieves over 99% recognition accuracy on the two datasets. Meanwhile, the proposed model provides better generalization performance and recognition accuracy than existing fault diagnosis methods.

## 1. Introduction

Rolling bearings are critical components in rotary machinery, widely used across various fields, including vehicles, aerospace, CNC machine tools, electromechanical equipment, and precision instruments. Often referred to as the “joints of industry”, rolling bearings are valued for their complex structure, high operating speeds, and excellent load-carrying capacity [1]. Moreover, its health status will directly affect the performance and safety of mechanical equipment. Bearing faults usually cause abnormal vibration and generate shocks within the mechanical system, threatening the equipment’s safety and productivity. Therefore, developing practical intelligent diagnostic technology to detect bearing faults in a timely manner is of great significance for preventing accidents and improving operational efficiency [2,3,4].

With the rapid development of deep learning (DL), its application in bearing fault diagnosis is becoming increasingly widespread [5]. DL can automatically extract features from collected data and directly link the raw monitoring data to the health state of bearings, significantly improving the efficiency and accuracy of fault diagnosis. Among DL, convolutional neural networks (CNNs), long short-term memory networks (LSTMs) [6], deep belief networks (DBNs) [7], and recurrent neural networks (RNNs) [8] are the most widely applied. Compared to traditional methods, these approaches can adaptively extract features from data without requiring expert knowledge and experience. This not only enhances diagnostic accuracy but also reduces the uncertainty introduced by human intervention [9,10]. However, many current intelligent diagnostic methods rely on the availability of sufficient labeled fault data to perform well. This presents challenges for the application of DL, as mechanical equipment typically operates in a normal state for extended periods [11]. The difficulty in obtaining fault samples ultimately leads to an imbalance between the number of normal and fault samples. This imbalance inhibits the precision and generalization ability of DL methods and hinders the development of DL techniques in fault diagnosis. Therefore, addressing the data imbalance problem is critical for the effective implementation and adoption of intelligent fault diagnosis.

Data augmentation is an effective method for addressing data imbalance problems [12]. Generative adversarial networks (GANs) are a widely used data augmentation model. However, GANs present problems such as instability, vanishing gradients, and pattern collapse during training. Consequently, researchers have invested significant effort in improving the training process and enhancing the quality of data generation. Arjovsky et al. [13] proposed Wasserstein generative adversarial networks (WGANs), which use the Wasserstein distance instead of Jensen–Shannon (J-S) divergence as a metric to quantify the difference between the generated and real data distributions, theoretically resolving the problem of training instability. However, WGANs can occasionally suffer from convergence issues and low-quality samples. To mitigate this, Gulrajani et al. [14] improved the WGAN loss function by replacing weight clipping with a gradient penalty, thereby enhancing model stability. Although improved GAN models can generate high-quality samples with relatively stable training, the adversarial nature of the training process between the generator and discriminator inherently introduces instability. Additionally, the parameters of both the generator and the discriminator need to be carefully tuned to prevent one network from becoming too powerful, which could lead to unstable training or even a failure to converge. Finally, since GANs are trained through complex adversarial processes, it remains difficult to explain their internal mechanisms and generation processes.

In recent years, diffusion models (DMs) have made a significant impact on generative tasks [15]. The DM is a generative model that consists of two main parts: a forward process and a reverse process. It first samples Gaussian noise from a prior distribution and then gradually removes the noise through the learned noise distribution. Through these processes, the DM can progressively generate high-quality samples from the initial noise distribution. The model’s parameters are optimized using maximum likelihood estimation to generate data that match the true distribution. The diffusion model not only has a solid theoretical foundation but also surpasses GANs in image synthesis [16].

On the other hand, the Transformer is a neural network with a global feature extraction capability, widely used in the field of fault diagnosis [17]. Yang et al. [18] proposed a bearing fault diagnosis method based on the Transformer neural network, which encodes the original vibration signals both linearly and positionally through the attention mechanism. Even without data preprocessing, this method achieves high diagnostic accuracy. Chu et al. [19] redesigned the spatial attention mechanism of the Transformer to improve model performance. Liu et al. [20] proposed the Swin Transformer model, which successfully addresses the differences between the image and text domains by introducing a hierarchical structure and a shift-window self-attention mechanism. The model performs well in tasks such as image classification, object detection, and segmentation, demonstrating the potential of the Transformer’s application in the field of image recognition.

In summary, existing generative models still face challenges related to instability and difficulty in convergence during the training process, while current fault diagnosis models exhibit limitations in capturing data features. Moreover, small sample sizes and sample imbalance remain pressing issues in the field of bearing fault diagnosis. To address these challenges, this paper proposes a novel bearing fault diagnosis method that combines denoising diffusion implicit modeling (DDIM) with the TransNet network.

First, the one-dimensional bearing signal data are transformed into two-dimensional images using the gram angle difference field (GADF). The DDIM is then employed to deeply learn the fault sample features, thereby expanding the small-sample dataset. This effectively mitigates the sample imbalance issue and reduces the risk of overfitting during small-sample training. Additionally, DDIM’s generative capability enhances the model’s ability to learn fault features, improving its performance under data-scarce conditions. Subsequently, the TransNet network model is applied for fault diagnosis on the expanded dataset, fully leveraging the temporal and spatial distribution features of bearing fault signals. This enhances both the robustness and generalization ability of the proposed method.

The experimental results demonstrate that the proposed method achieves over 99% recognition accuracy on both the CWRU dataset and the Nanchang Railway Bureau dataset, significantly outperforming other comparative models. These findings validate the effectiveness and practicality of the proposed approach. The main contributions of this paper are as follows:

(1) The one-dimensional vibration signal of a bearing is converted into a two-dimensional image by using the Gramian angular difference field. This transformation helps convolutional neural networks extract features at multiple scales, from local to global, including subtle edges and textures to overall patterns and shapes. Additionally, the method can directly capture the time–frequency characteristics of the signal and the spatial correlations between neighboring data points, thereby effectively improving the accuracy of fault diagnosis.
(2) The DDIM is used to expand the bearing dataset, addressing the small-sample problem. By generating high-quality samples that resemble the distribution of the original data, this approach not only improves classification accuracy but also enhances the generalization ability of the model. Moreover, DDIM preserves the diversity of fault features, helping the model better adapt to different types of faults, which in turn accelerates training convergence and improves overall model performance.
(3) A deep learning model that integrates convolutional neural networks with Transformers is proposed. This model effectively combines deep, multi-scale features. The integration of CNNs’ latent feature extraction with the Transformer encoder’s attention mechanism significantly enhances the robustness and generalizability of the image classification system.

The remainder of this paper is organized as follows. Section 2 presents the underlying theory of this paper. Section 3 provides details of the proposed TransNet method, including its basic components and the fault diagnosis process. In Section 4, the effectiveness and superiority of the TransNet method are verified using the CWRU bearing dataset and the Nanchang Railway Bureau dataset. The conclusions are presented in Section 5.

## 2. Preliminaries

### 2.1. Gramian Angular Difference Fields

The Gram angular difference field (GADF) is an effective method for converting one-dimensional time series data into two-dimensional images [21]. This approach not only preserves the original information of the signal but also effectively retains the time dependence of the time series data. By calculating the angular difference between different time points in the series, a two-dimensional matrix is generated that describes the trend of the data. This matrix converts the one-dimensional data into a two-dimensional image, allowing algorithms and techniques from the field of image processing to be applied for feature extraction and pattern recognition. The specific process is as follows:

(1) Assume that the given time series is $X=\{x_{1},x_{2},\cdots ,x_{n}\}$, where $x_{i}$ is the *i*-th sample signal, and i=1,2,…,n is the number of sampling points. To ensure that the inner product value is not biased towards the maximum value in the sequence, the sequence is mean normalized using Equation (1), and the sequence values are scaled to the range [−1, 1].

(1) $${\tilde{x}}_{i}=\frac{\left[x_{i}-max(X)]+[x_{i}-min(X)\right]}{max(X)-min(X)}$$

(2) Then, polar coordinates are used to represent the normalized time series. It can be expressed as follows:

(2) $$\left\{\begin{array}{l} \phi =\arccos({\tilde{x}}_{i}),-1\leq {\tilde{x}}_{i}\leq 1,{\tilde{x}}_{i}\in \tilde{X}\\ r=\frac{t_{i}}{N},t_{i}\in N \end{array}\right.$$

where ϕ denotes the cosine of the coding angle, $t_{i}$ is the timestamp, r is the radius that maintains time dependence, and *N* is the constant factor of the regularization system. The entire encoding process is bijective, ensuring the integrity of the information.

(3) The Gram matrix represents the inter-relationships between features and the correlations among features and dimensions. Multi-scale representation information can be obtained through the inner product operation. The main diagonal elements of the matrix reflect the attributes of the features themselves, while the other elements demonstrate the strong connections between different features. The Gramian angle field that follows the transformation of the signal sequence to the polar coordinate system is defined as follows:

(3) $$\begin{array}{c} G=\left[\begin{array}{cccc} \cos(\phi_{1}+\phi_{1}) & \cos(\phi_{1}+\phi_{2}) & \cdots & \cos(\phi_{1}+\phi_{n})\\ \cos(\phi_{2}+\phi_{1}) & \cos(\phi_{2}+\phi_{2}) & \cdots & \cos(\phi_{2}+\phi_{n})\\ \vdots & \vdots & \cdots & \vdots \\ \cos(\phi_{n}+\phi_{1}) & \cos(\phi_{n}+\phi_{1}) & \cdots & \cos(\phi_{n}+\phi_{n}) \end{array}\right]\\ ={\tilde{X}}^{\prime }\cdot \tilde{X}-{\sqrt{I-{\tilde{X}}^{2}}}^{\prime }\cdot \sqrt{I-{\tilde{X}}^{2}} \end{array}$$

where I is the unit row vector, and ${\tilde{X}}^{\prime }$ is the transpose of $\tilde{X}$.

### 2.2. Denoising Diffusion Implicit Models

Denoising diffusion implicit models (DDIMs) [22] are state-of-the-art generative models designed to generate high-quality images or other types of data. DDIMs are an improvement upon denoising diffusion probabilistic models (DDPMs), combining the advantages of implicit generative models while enhancing generation efficiency without compromising quality. Denoising diffusion consists of two processes: the forward propagation process and the backward propagation process.

#### 2.2.1. Forward Diffusion Process

The forward diffusion process is a step-by-step transformation of a raw signal (e.g., a vibration signal or an image) into pure noise. The process is based on a given time step, on which noise is gradually added to the data and, finally, the signal becomes almost random Gaussian noise. Since the state transition at a given moment in the forward propagation process depends only on the state at the previous moment, the state transition at a given moment can be represented by a conditional probability distribution $Q(X_{t}|X_{t-1})$. $Q(X_{t}|X_{t-1})$ can be calculated by

(4) $$Q(X_{t}|X_{t-1})∼N(\sqrt{1-\beta_{t}}X_{t-1},\beta_{t}I)$$

where N is a Gaussian distribution with mean $\sqrt{1-\beta_{t}}X_{t-1}$ and covariance $\beta_{t}I$, and $\beta_{t}$ is the variance of the noise corresponding to the time step t, controlling the amount of noise added at each step.

In each step, the introduced noise is an independent Gaussian noise. To quantify the cumulative effect of adding noise at each step, a cumulative noise coefficient $\alpha_{t}$ is defined:

(5) $$\alpha_{t}=\overset{t}{\underset{s=1}{\prod }}\left(1-\beta_{s}\right)$$

where $\alpha_{t}$ denotes the proportion of the original component of the signal at time step *t*. As *t* increases, $\alpha_{t}$ becomes smaller and smaller, indicating that the weight of the original signal is gradually being overtaken by noise.

To simplify the recursive computation, the DDIM calculates the signal $X_{t}$ at each time step by directly combining the original signal $X_{0}$ with a linear combination of random noise, thus making it no longer a Markov process. The formula is as follows:

(6) $$X_{t}=\sqrt{\alpha_{t}}X_{0}+\sqrt{1-\alpha_{t}}Z_{t}$$

where $Z_{t}$ is random noise.

The forward propagation process requires a training model that predicts the noise $Z_{0}$ at time step *t* for the backpropagation process. $X_{t}$ consists of the original vibration signal and the added noise, which is one of the input model parameters.

#### 2.2.2. Reverse Generation Process

The inversion process begins with a random noisy signal $X_{t}$, which is progressively denoised until it is eventually restored to a state close to the original data $X_{0}$. This inversion process is designed to be deterministic. The backpropagation process is also deterministic, and its propagation is structured as an implicit model. In this model, a system is trained to predict the noise at each step and recover a clean signal from the noise. The backpropagation process can be calculated by

(7) $$X_{t-1}=\sqrt{\alpha_{t-1}}X_{0}+\sqrt{1-\alpha_{t-1}}Z_{t}$$

where $Z_{t}$ is the noise predicted from the noise of the current step *t*. To predict $Z_{t}$, a denoising model is required to estimate the noise distribution at each step *t*. At this point, the backpropagation formula can be expressed as follows:

(8) $$X_{t-1}=\frac{1}{\sqrt{\alpha_{t-1}}}\left(X_{t}-\sqrt{1-\alpha_{t-1}}\varepsilon_{\theta }(X_{t},t)\right)$$

where $\varepsilon_{\theta }(X_{t},t)$ is the noise predicted by the denoising model, and $\alpha_{t-1}$ is the cumulative noise coefficient for the time step t−1. The denoising model $\varepsilon_{\theta }$ is trained by modeling the noise in the forward process. The training objective of the model is to minimize the difference between the predicted noise and the actual noise. Mathematically, it can be expressed as

(9) $$L_{\mathrm{noise}}=E_{X_{0},Z_{t},t}\left[\varepsilon_{\theta }(X_{t},t)-{Z_{t}}^{2}\right]$$

### 2.3. Transformer Encoders

The Transformer encoder layer is the core component of the Transformer model architecture [23], responsible for processing input sequences and generating representation vectors that contain contextual information. Each encoder layer consists of two main sublayers: the multi-head attention and the feedforward neural network (FFN). Additionally, each sublayer is followed by layer normalization and a residual connection.

The multi-head self-attention mechanism allows the model to capture global contextual dependencies by simultaneously focusing on information from different locations in the input sequence. In this way, the model is able to capture various features from the inputs and form a rich feature map from the connected outputs of these features. The relevant formulas are shown in (10)–(12).

(10) $$\mu (Q,K,V)=\mathrm{Concat}({\mathrm{head}}_{1},\ldots ,{\mathrm{head}}_{h})W^{O}$$

(11) $${\mathrm{head}}_{i}=\mathrm{Attention}(QW_{i}^{Q},KW_{i}^{K},VW_{i}^{V})$$

(12) $$\mathrm{Attention}(Q,K,V)=\mathrm{softmax}\left(\frac{QK^{T}}{\sqrt{d_{k}}}\right)V$$

where Q, *K*, and *V* represent queries, keys, and values, respectively. $W_{i}^{Q}$, $W_{i}^{K}$, and $W_{i}^{V}$ are the weight matrices of each attention head. $d_{k}$ is the dimension of the key, and $W^{O}$ is the weight matrix of the output of the attention head. $QK^{T}$ is used to compute the dot-product similarity between the query and the key, which measures the correlation between different positions in the sequence. This similarity is then normalized into a probability distribution using the softmax function. Finally, the weighted sum of the normalized weights is used to output a vector that captures global dependencies.

The output of the multi-head self-attention mechanism is added to the residual connection of the input sequences and then layer normalized. Mathematically, it can be expressed as

(13) $$Output_{Attention}=LayerNorm(X+MultiHead(Q,K,V))$$

Since the attention mechanism, the Transformer’s encoder layer, also contains a simple FFN that performs the same nonlinear transformation on each input position vector individually. This FFN consists of two linear transformations with a GELU activation function in between. The FFN can be calculated by

(14) $$\mathrm{FFN}(\mu_{o})=\mathrm{GELU}(0,X\times W_{1}+b_{1})W_{2}+b_{2}$$

where X is the input to the FNN, $W_{1}$ and $W_{2}$ are the weight matrices, and $b_{1}$ and $b_{2}$ are the bias vectors of the FNN. The output of the feedforward neural network is similarly normalized by residual connections and layer normalization. Mathematically, it can be expressed as

(15) $$Output_{FNN}=LayerNorm(Output_{Attention}+FNN(Output_{Attention}))$$

The final encoder output can be expressed as

(16) Output=LayerNorm(Attention(LayerNorm(X))+FNN(LayerNorm(X)))

## 3. Proposed Method

### 3.1. Model Architecture

**Figure 1:**
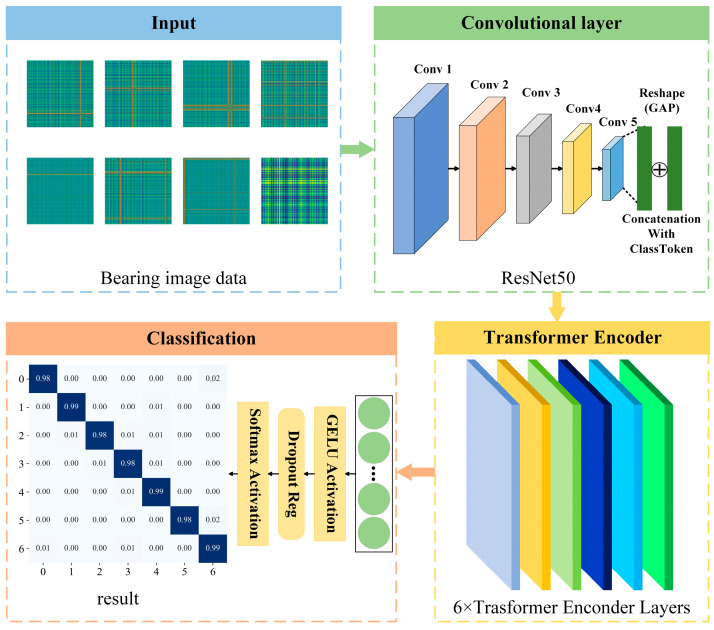
The TransNet model proposed in this paper.

In this paper, we propose an innovative framework and introduce a unified model that cleverly combines the capabilities of convolutional neural networks in complex feature recognition with the spatial understanding capabilities of the Transformer architecture. Specifically, the unified model uses ResNet50 [24] as the backbone network, followed by a Transformer encoder and finally equipped with a classifier. In addition, a specially designed Class Token layer is added to provide a global contextual link for the local characterization of the model. The proposed TransNet model consists of three main components: a CNN convolutional block, a Transformer encoder, and a classifier, as shown in Figure 1.

### 3.2. Fault Diagnosis Based on TransNet

**Figure 2:**
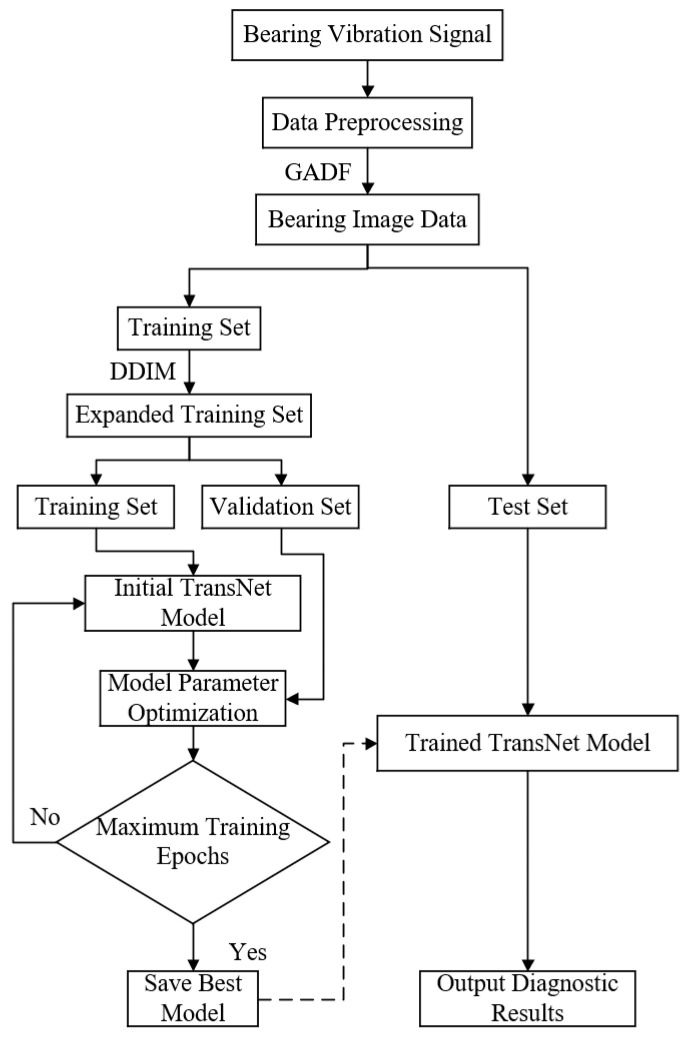
Fault diagnosis flowchart based on TransNet.

**Figure 3:**
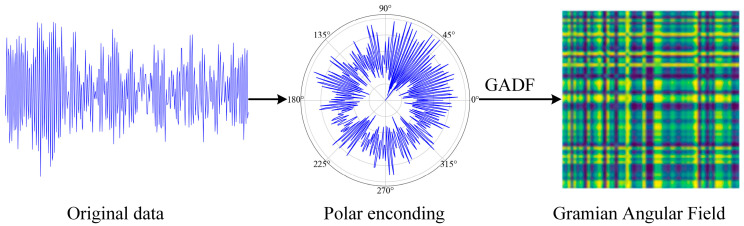
Original data are converted into 2D images by GADF.

The fault diagnosis process in this paper is shown in Figure 2. First, the bearing vibration signals are normalized to eliminate scale differences in the signals. Then, the normalized time series is represented using polar coordinates according to Equation (2). Finally, the multi-scale representation information is generated according to Equation (3) and the inner product operation to form a 2D image, and the specific process is illustrated in Figure 3. The converted image data are proportionally divided into a training set and a test set, and the training set undergoes the reverse denoising process of the DDIM to generate new samples for data enhancement. The expanded bearing fault images are then input into the TransNet network for training, and the model parameters with the best results are saved. Finally, the test set is input into the trained model to obtain the fault diagnosis results.

## 4. Experimental Validation and Analysis

In this paper, the diagnostic performance of the model is verified using the CWRU bearing dataset and the real bearing dataset from the locomotive depot of the Nanchang Railway Bureau. The test running environment is TensorFlow-GPU 2.6.0. All the experiments are carried out on a PC with AMD Ryzen 7 3700X CPU, 32 GB RAM, and NVIDIA GeForce RTX 2080Ti GPU. Table 1 provides a brief parameters overview of the TransNet. The other settings are as follows:

(1) The Adam optimizer is used, and the initial learning rate is set to 0.0001.

(2) A cosine decay learning rate scheduler is implemented, where the learning rate undergoes a cosine decay every 10,000 training steps. This approach helps improve the stability of model training and convergence speed.

(3) The use of mixed-precision training helps to reduce the computational demand during the training process.

To mitigate the effect of randomness, each experiment is conducted 10 times, with the average result taken as the final outcome.

### 4.1. Case 1: Experimental Verification Based on CWRU Dataset

**Figure 4:**
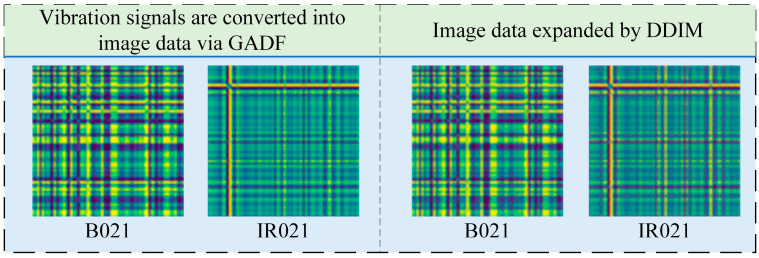
Part of the image data from the CWRU dataset.

The experimental data were obtained from the acceleration dataset of rolling bearings at the drive end, provided by the bearing data center at Case Western Reserve University (CWRU) [25]. The acceleration data at the drive end of the test stand were selected. The test stand mainly consists of a motor, a torsion encoder, a power meter, and control electronics, with a sampling frequency of 12 kHz and a rotational speed of 1797 r/min. The dataset comprises three types of faults: rolling element faults, outer ring faults, and inner ring faults, which are generated by EDM machining. The fault levels are 0.007 in, 0.014 in, and 0.021 in, resulting in a total of nine different fault types. The vibration signals are converted into image data using the GADF, with 300 fault samples obtained for each type. The training set and validation set are divided at a 4:1 ratio, and the DDIM is used to expand the training set to 540 samples for each type. Some of the image data are shown in Figure 4. The construction of the sample set is shown in Table 2.

#### 4.1.1. Experimental Results of the TransNet Model for Fault Diagnosis in Case 1

**Figure 5:**
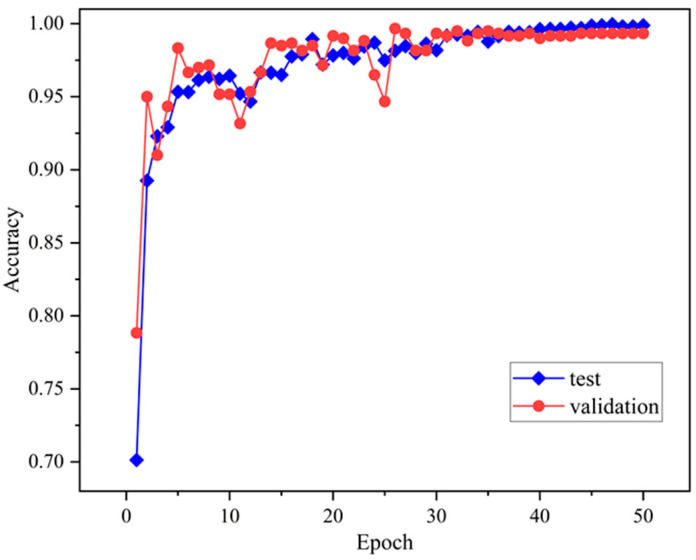
Accuracy variation curve.

Figure 5 demonstrates the results of the TransNet model proposed in this paper for the CWRU bearing dataset after 50 rounds of iterative training. Before training, to address the small-sample problem and enhance the model’s generalization ability, this paper adopts the DDIM method to augment the sample data. The analysis shows that the model quickly improves both training and validation accuracy in the early stages (the first five epochs), demonstrating its strong ability to learn data features. As the training progresses, the accuracies of both the training set and the validation set gradually stabilize and approach 1.0, which fully demonstrates that the model exhibits excellent classification performance in both the training and validation phases.

**Figure 6:**
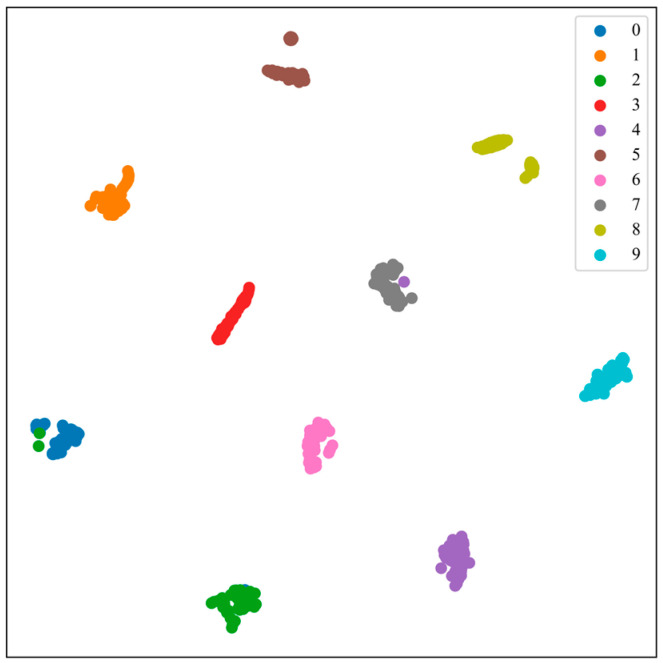
Clustering results for Case 1.

To further validate the performance of the model, the optimal TransNet model obtained during the training process was evaluated on the test set, and the confusion matrix is presented in Figure 6. The results indicate that most categories achieve high classification accuracy, including categories B3, B5, B6, B8, and B9, all of which achieve 100% correct classification. One sample in B0 is misclassified as B2, one sample in B4 is also misclassified as B0, and two samples in B2 are misclassified as B0. This indicates some confusion between categories B0 and B2, which can be attributed to the similarity in fault characteristics after the vibration signals are converted to image data. Overall, the model proposed in this paper exhibits robust classification performance.

Feature visualization through T-SNE allows for further observation of the feature extraction capability and classification performance of the model. As shown in Figure 6, most categories form clear clusters in the 2D space, indicating that the model has strong feature extraction and category differentiation abilities, particularly in distinguishing normal categories from other fault categories. Only a small number of samples from individual categories (e.g., B2 and B4) are misclassified; however, overall, the model remains capable of distinguishing between these categories.

#### 4.1.2. Comparison Experiments in Case 1

In order to verify the performance of the TransNet model and the superiority of its methodology, this paper designs comparative experiments. The unprocessed ResNet50, ResNet101, and VGG16 models are selected as benchmark models to ensure that the experiments are representative and comparable. All models were rigorously trained and tested on the CWRU 10-class bearing fault image dataset, and the experimental conditions were kept consistent to eliminate interference from external factors.

**Figure 7:**
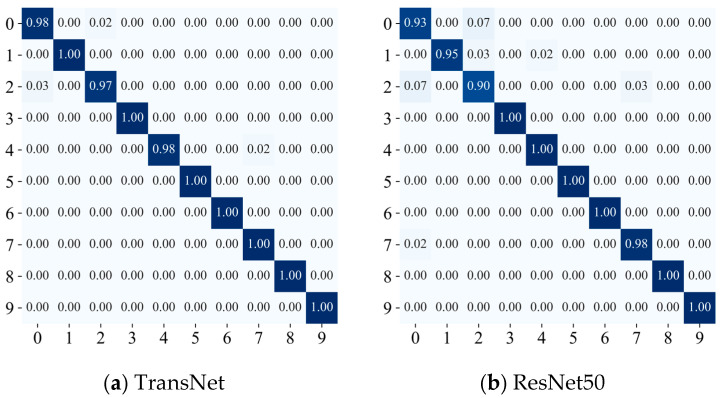

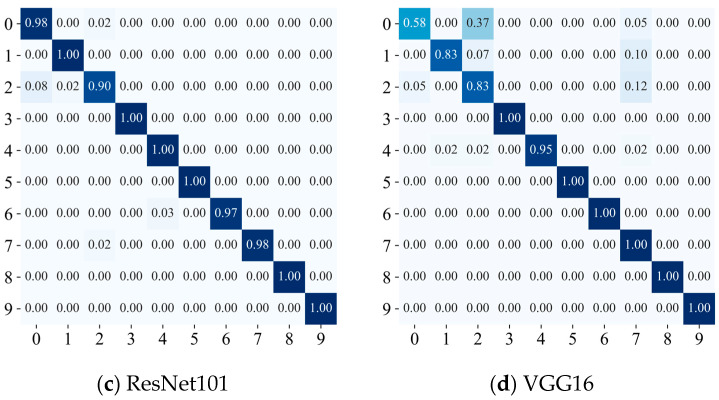
Confusion matrix for Case 1.

**Figure 8:**
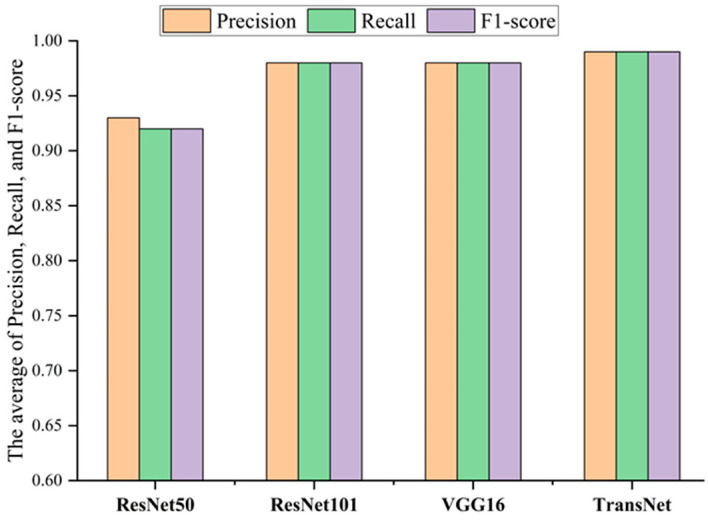
Comparison of fault diagnosis results among different methods.

Meanwhile, to minimize the impact of randomness associated with a single experiment, this paper conducts a total of four groups of experiments, with each group containing ten classification trials. The final results are presented as the average of these trials. The comparison results are illustrated in Figure 7 and Figure 8, highlighting the differences in classification performance and accuracy between the TransNet model and the unprocessed ResNet50, ResNet101, and VGG16 models. The experimental results show that the classification effects of TransNet model are all better than other models, and all evaluation indexes have achieved better scores, which further verifies the superiority and effectiveness of this paper’s method in bearing fault diagnosis.

### 4.2. Case 2: Experimental Verification Based on Nanchang Railway Bureau Dataset

**Figure 9:**
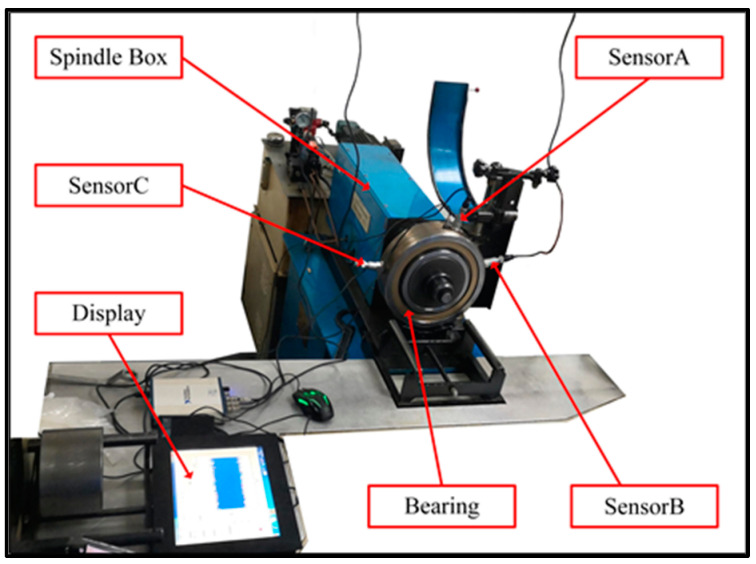
Test bench for locomotive bearings at Nanchang Railway Bureau.

The locomotive bearing test bench of the Nanchang Railway Bureau is shown in Figure 9. It is mainly composed of the spindle box, hydraulic system, electrical system, and table body. In the spindle box, the bearing to be tested is installed for detection, rotation, radial loading, and other processes. The hydraulic system is responsible for applying radial loading and unloading to the bearing during testing. The power supply is 380 V, 50 Hz, and a three-phase four-wire alternating current, with a total motor drive power of 5 kW. The test rotational speed is set at 500 rpm. The locomotive bearings used in the test are the NJ2232WB series of cylindrical roller bearings, with an inner diameter of 160 mm and an outer diameter of 290 mm. The vibration signals of the bearings are collected using three acceleration sensors (A, B, and C) at a sampling frequency of 20 kHz. The six types of locomotive bearing vibration signal data collected by sensor B include normal bearing, three different degrees of inner ring failure, two different degrees of outer ring failure, and cage failure. These are sequentially numbered as C0 to C6. Compared to Case 1, the dataset size in Case 2 is different; however, the experimental steps are largely the same, and the construction of the data samples is similar. The construction of the data samples is shown in Table 3.

#### 4.2.1. Experimental Results of the TransNet Model for Fault Diagnosis in Case 2

**Figure 10:**
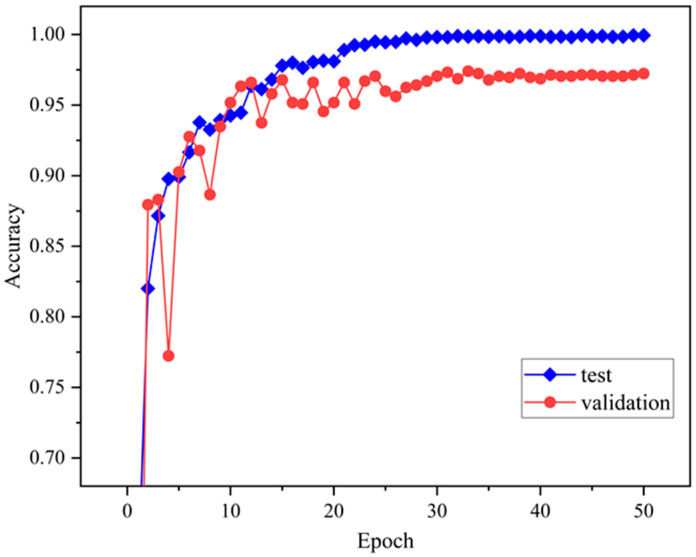
Change curve of recognition accuracy.

As shown in Figure 10, the accuracy of the model gradually improves and stabilizes as the number of training rounds increases. The accuracy of the training set quickly approaches 1.0, while the accuracy of the validation set remains at a high level after slight fluctuations. This indicates that the model not only performs well on the training data but also exhibits good generalization ability on the validation set.

The small-sample problem is addressed through DDIM expansion. The DDIM method effectively increases the number of samples and enhances the model’s learning ability in small-sample data scenarios, thereby improving classification performance. The experimental results indicate that the model exhibits greater robustness and stability after data expansion, providing reliable support for the bearing fault diagnosis task. This finding further validates the effectiveness of the method proposed in this paper under small-sample conditions.

**Figure 11:**
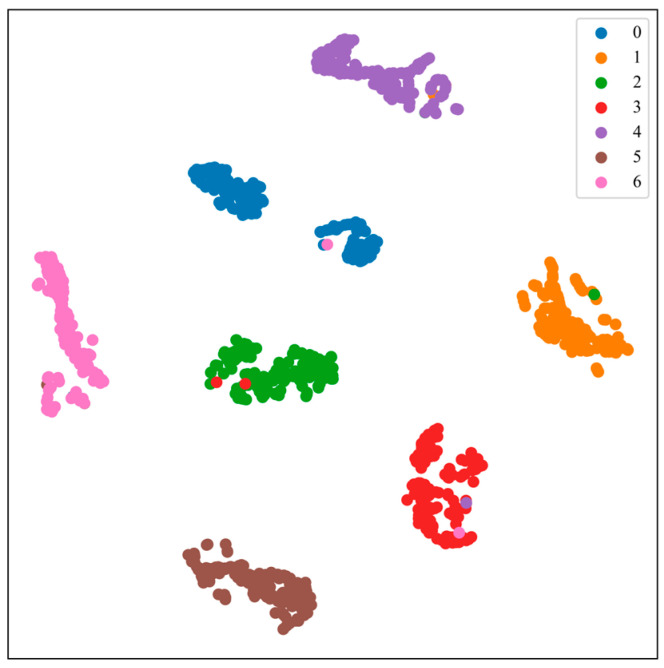
Clustering effect diagram of Case 2.

As shown in Figure 11, the different bearing fault categories exhibit distinct clustering distributions in the two-dimensional space, demonstrating the excellent performance of the TransNet model proposed in this paper in feature extraction. In particular, the distinction between normal categories (e.g., C0) and fault categories is clearer, indicating that the model can effectively recognize bearing signals in different states. However, the sample distribution of certain categories (e.g., C6) is slightly dispersed, indicating high feature similarity among some categories, which may result in classification confusion.

In conclusion, the t-SNE clustering diagram clearly demonstrates the model’s ability to extract features and differentiate categories, further validating the effectiveness and robustness of the method proposed in this paper for bearing fault diagnosis.

**Figure 12:**
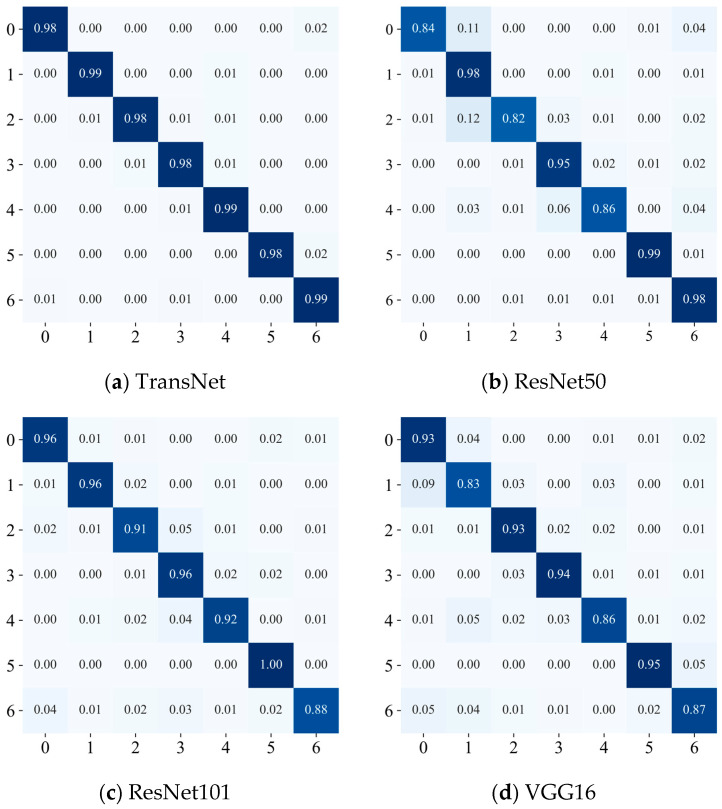
Confusion matrix of Case 2.

The saved model is evaluated against the test set, and the confusion matrix is presented in Figure 12a. The results indicate that the model performs very well in the classification task. The diagonal entries of the matrix predominantly reflect high accuracy classification results, suggesting that the model can accurately recognize most categories. Although individual categories, such as C3 and C6, are occasionally confused, the overall classification accuracy remains very high, reaching or even exceeding 98% for most categories.

In addition, the confusion matrix indicates that the model performs well in distinguishing between different classes of bearing faults. After expanding the samples using DDIM, the model effectively mitigates the challenges associated with small sample sizes and further enhances its generalization performance. These results fully validate that the TransNet network architecture proposed in this paper demonstrates significant advantages in the bearing fault diagnosis task.

#### 4.2.2. Comparison Experiments in Case 2

**Figure 13:**
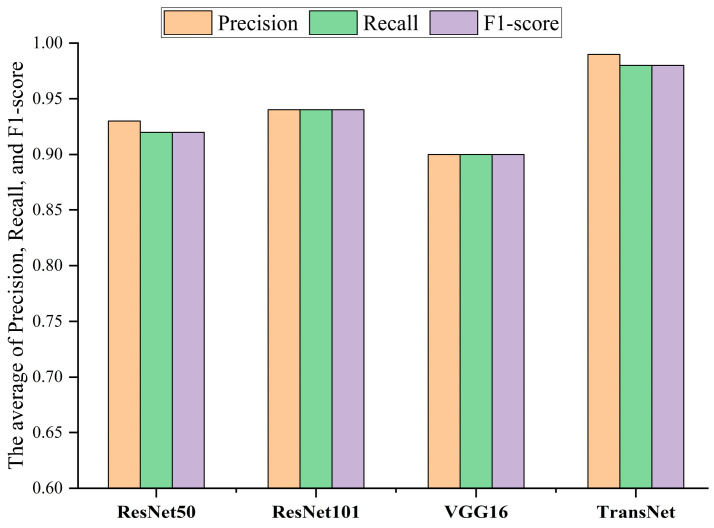
Comparison of fault diagnosis among different methods.

Comparative experiments are conducted to evaluate the performance of the TransNet model and demonstrate the superiority of its approach. The unprocessed ResNet50, ResNet101, and VGG16 are selected as benchmark models. All models were rigorously trained and tested on the seven-class bearing fault image dataset from the Nanchang Railway Bureau, and the results of the comparison experiments are presented in Figure 12 and Figure 13. The experimental results show that the overall classification effect of the TransNet model is much better than that of other models, and other models have large classification errors in some categories, while TransNet has 98% classification results in all categories. All indicators are also better than other benchmark models, further verifying the superiority and effectiveness of this paper’s method in bearing fault diagnosis.

## 5. Conclusions

Aiming to improve the data imbalance problem in bearing fault diagnosis, this paper proposes a fault diagnosis framework based on the DDIM data enhancement method combined with the TransNet model. The conclusions are as follows:

(1) The conversion of one-dimensional vibration signals of bearings into two-dimensional images via GADF enables the CNNs to extract features locally and globally, capturing both subtle edges and textures and overall patterns and shapes. This transformation also allows the time–frequency characteristics of the signal and the spatial correlation of neighboring data points to be directly captured, thereby enhancing the accuracy of fault diagnosis.

(2) The data imbalance problem is addressed by utilizing the DDIM to expand the bearing dataset. By generating high-quality samples that resemble the original data distribution, classification accuracy and generalization ability are improved. Moreover, this approach preserves the diversity of fault features, helping the model better adapt to different types of faults, ultimately accelerating training convergence and enhancing the model’s overall performance.

(3) A deep learning model integrating CNNs and Transformer is proposed, combining deep and complex multi-scale features. The potential feature extraction capability of CNNs is innovatively combined with the attention mechanism of the Transformer encoder, significantly enhancing the robustness and generalizability of the image classification system.

(4) The model was validated and analyzed using the CWRU bearing dataset and the Nanchang Railway Bureau dataset. The results show that the adopted DDIM can effectively alleviate the sample imbalance problem and reduce the risk of overfitting in small-sample training. Meanwhile, the recognition accuracy of the proposed TransNet on both datasets exceeds 99%, demonstrating better generalization performance and recognition accuracy compared to other existing fault diagnosis methods.

(5) This paper mainly focuses on fault diagnosis under a single operating condition. Future research will explore fault diagnosis technologies under more complex conditions, such as variable load and cross-equipment scenarios, to provide strong support for the health management and maintenance of industrial equipment. Additionally, while this paper is primarily based on data-driven research, future work will analyze the mechanisms behind bearing fault characteristics to further deepen the research on bearing fault diagnosis.

## Figures and Tables

**Table 1:** Summary of parameters distribution in TransNet Architecture.

Layer Group	Output Shape	Param
Input and Initial Convolution	(112, 112, 64)	9728
Residual Blocks (Conv2_x)	(56, 56, 256)	220032
Residual Blocks (Conv3_x)	(28, 28, 512)	1230336
Residual Blocks (Conv4_x)	(14, 14, 1024)	7129088
Residual Blocks (Conv5_x)	(7, 7, 2048)	14998528
Transformer Layers and Class Token	(2, 2048)	310556672
Final Dense Layer	(10)	40907

**Table 2:** Fault samples from the CWRU bearing dataset.

Fault Type	Fault Size/in	Original Training Set	Expanded Training Set	Test Set	Label
Inner Ring Fault	0.007	240	540	60	B0
	0.014	240	540	60	B1
	0.021	240	540	60	B2
Rolling Element Fault	0.007	240	540	60	B3
	0.014	240	540	60	B4
	0.021	240	540	60	B5
Outer Ring Fault	0.007	240	540	60	B6
	0.014	240	540	60	B7
	0.021	240	540	60	B8
Normal	/	540	540	60	B9

**Table 3:** Fault samples of the locomotive bearing dataset from Nanchang Railway Bureau.

Fault Type	Fault Size/mm	Original Training Set	Expanded Training Set	Test Set	Label
Inner Ring Fault	4	720	1620	180	C0
	7	720	1620	180	C1
	19	720	1620	180	C2
Outer Ring Fault	7	720	1620	180	C3
	19	720	1620	180	C4
Rolling Element Fault	4	720	1620	180	C5
Normal	/	1620	1620	180	C6

## Data Availability

The dataset is available on request from the authors.

## References

[1] Zhang J., Sun Y., Guo L., Gao H., Hong X., Song H. (2020). A new bearing fault diagnosis method based on modified convolutional neural networks. Chin. J. Aeronaut..

[2] Pang B., Liu Q., Xu Z., Sun Z., Hao Z., Song Z. (2024). Fault vibration model driven fault-aware domain generalization framework for bearing fault diagnosis. Adv. Eng. Inform..

[3] Hakim M., Omran A.A.B., Ahmed A.N., Al-Waily M., Abdellatif A. (2023). A systematic review of rolling bearing fault diagnoses based on deep learning and transfer learning: Taxonomy, overview, application, open challenges, weaknesses and recommendations. Ain Shams Eng. J..

[4] Liu J., Wang C., Wang R., Xiao Q., Wang X., Wu S., Zhang L. (2024). A Novel Multiscale Adaptive Graph Adversarial Network for Mechanical Fault Diagnosis. Knowl.-Based Syst..

[5] Neupane D., Seok J. (2020). Bearing fault detection and diagnosis using case western reserve university dataset with deep learning approaches: A review. IEEE Access.

[6] Chen X., Zhang B., Gao D. (2021). Bearing fault diagnosis base on multi-scale CNN and LSTM model. J. Intell. Manuf..

[7] Pan Y., Cheng D., Wei T., Jia Y. (2022). Rolling bearing performance degradation assessment based on deep belief network and improved support vector data description. Mech. Syst. Signal Process..

[8] Choi J., Lee S.J. (2023). RNN-based integrated system for real-time sensor fault detection and fault-informed accident diagnosis in nuclear power plant accidents. Nucl. Eng. Technol..

[9] He M., He D. (2017). Deep learning based approach for bearing fault diagnosis. IEEE Trans. Ind. Appl..

[10] Liu J., Wan L., Xie F., Sun Y., Wang X., Li D., Wu S. (2024). Cross-machine deep subdomain adaptation network for wind turbines fault diagnosis. Mech. Syst. Signal Process..

[11] Xu K., Kong X., Wang Q., Han B., Sun L. (2023). Intelligent fault diagnosis of bearings under small samples: A mechanism-data fusion approach. Eng. Appl. Artif. Intell..

[12] Ruan D., Chen X., Gühmann C., Yan J. (2023). Improvement of generative adversarial network and its application in bearing fault diagnosis: A review. Lubricants.

[13] Arjovsky M., Chintala S., Bottou L. Wasserstein generative adversarial networks. Proceedings of the International Conference on Machine Learning, PMLR.

[14] Gulrajani I., Ahmed F., Arjovsky M., Dumoulin V., Courville A.C. (2017). Improved training of wasserstein gans. Adv. Neural Inf. Process. Syst..

[15] Chen M., Mei S., Fan J., Wang M. (2024). Opportunities and challenges of diffusion models for generative AI. Natl. Sci. Rev..

[16] Dhariwal P., Nichol A. (2021). Diffusion models beat gans on image synthesis. Adv. Neural Inf. Process. Syst..

[17] Han K., Wang Y., Chen H., Chen X., Guo J., Liu Z., Tang Y., Xiao A., Xu C., Xu Y. (2022). A survey on vision transformer. IEEE Trans. Pattern Anal. Mach. Intell..

[18] Yang Z., Cen J., Liu X., Xiong J., Chen H. (2022). Research on bearing fault diagnosis method based on transformer neural network. Meas. Sci. Technol..

[19] Chu X., Tian Z., Wang Y., Zhang B., Ren H., Wei X., Xia H., Shen C. (2021). Twins: Revisiting the design of spatial attention in vision transformers. Adv. Neural Inf. Process. Syst..

[20] Liu Z., Lin Y., Cao Y., Hu H., Wei Y., Zhang Z., Lin S., Guo B. Swin transformer: Hierarchical vision transformer using shifted windows. Proceedings of the IEEE/CVF International Conference on Computer Vision.

[21] Wang Z., Zhang J., Xia Y., Chen P., Wang B. (2022). A general and scalable vision framework for functional near-infrared spectroscopy classification. IEEE Trans. Neural Syst. Rehabil. Eng..

[22] Song J., Meng C., Ermon S. (2020). Denoising diffusion implicit models. arXiv Prepr..

[23] Prezja F., Annala L., Kiiskinen S., Lahtinen S., Ojala T., Ruusuvuori P., Kuopio T. (2023). Improving performance in colorectal cancer histology decomposition using deep and ensemble machine learning. arXiv Prepr..

[24] Karthika S., Durgadevi M., Rani T.Y. (2024). Enhancing Diabetic Retinopathy Diagnosis with ResNet-50-Based Transfer Learning: A Promising Approach. Ann. Data Sci..

[25] Loparo K., Case Western Reserve University Bearing Data Center Bearings Vibration Data Sets, Case Western Reserve University, 2012: 22–28. https://engineering.case.edu/bearingdatacenter/download-data-file.

